# The large extracellular loop is important for recruiting CD63 to exosomes

**DOI:** 10.17912/micropub.biology.000842

**Published:** 2023-08-03

**Authors:** Daniel Ivanusic, Joachim Denner

**Affiliations:** 1 Sexually transmitted bacterial pathogens and HIV (FG18), Robert Koch Institute, 13353 Berlin, Germany; 2 Institute of Virology, Department of Veterinary Medicine, Free University Berlin, 14163 Berlin, Germany.

## Abstract

Exosomes are small extracellular vesicles that are secreted from cells. To characterize exosome fraction marker proteins of the tetraspanin family in particular, CD9, CD63, and CD81 are routinely used. CD63 expression constructs were employed to investigate the influence of the large extracellular loop (LEL) of CD63 on sorting into exosomes. When the LEL of CD63 fused with mCherry was deleted, the protein was no longer found in the purified exosome fraction. This finding demonstrates the importance of the LEL sequence for the recruitment of CD63 into exosomes.

**Figure 1. Influence of CD63 LEL on recruiting CD63 into exosomes:**
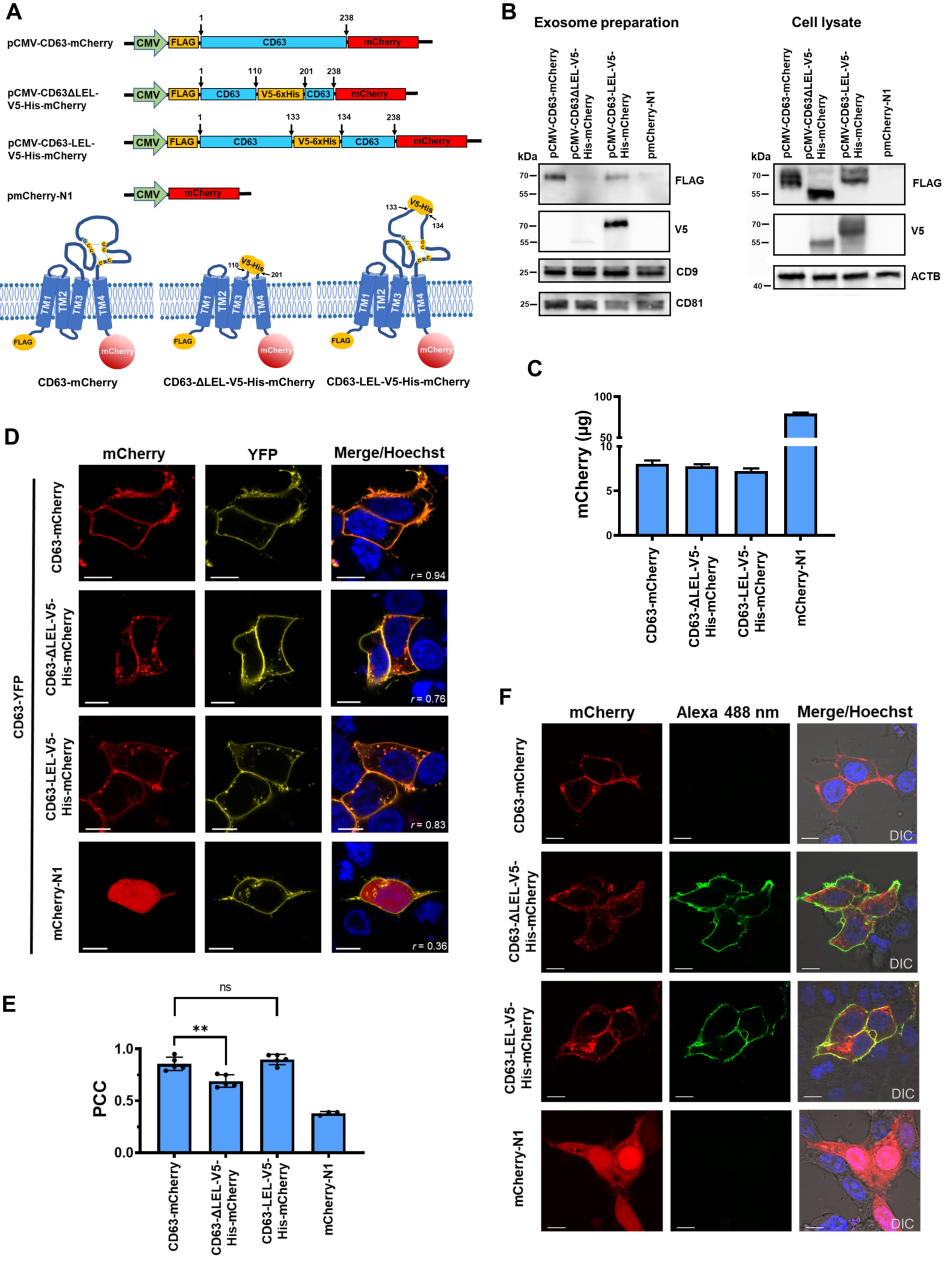
( **A** ) Schematic presentation (not to scale) of expression constructs and topology of expressed proteins. TM, transmembrane domain; C, cysteine; G, glycine; disulfide bridges are illustrated by broken lines. ( **B** ) Western Blot analysis of exosomes and transfected HEK293T cells 48 h post transfection. ACTB, actin beta. ( **C** ) Quantification of expressed mCherry and mCherry fused proteins from transfected HEK293T cells. The amount of mCherry was determined from cells of a well from a 6-well plate. Cells were analyzed 48 h post transfection and assayed in triplicate. ( **D** ) Representative confocal laser scanning microscopy (cLSM) images obtained from co-localization experiments using HEK293T cells. The Pearson correlation coefficient is displayed on each image. ( **E** ) Scatter plot diagrams of the Pearson correlation coefficient (PCC) calculated for several cLSM images. Student's t-test was used to verify the statistical significance between groups. ( **F** ) Demonstration of the extracellular accessibility of the V5-epitope. All scale bars, 10 µm, differential interference contrast (DIC), nuclei were stained with Hoechst 33342 (blue).

## Description

Exosomes are specified as spherical lipid bilayer vesicles with a diameter of 30–100 nm secreted from cells (Raposo and Stoorvogel, 2013; Willms et al., 2016) . They are considered small extracellular vesicles that are secreted from cells after fusion with the plasma membrane of specific endosomes called multivesicular bodies (MVB) (Hessvik and Llorente, 2018; Théry et al., 2018). Tetraspanin proteins are mainly used as marker proteins to characterize exosomes from fluid isolation. In particular, CD9, CD63, and CD81 are routinely used as exosome markers (Escola et al., 1998; Fordjour et al., 2022; Théry et al., 1999). The essential domain for recruitment of a tetraspanin into exosomes is not known. There are reports that in membranes CD63 and other tetraspanin proteins are organized in tetraspanin-enriched microdomains (TEMs). These highly organized membrane areas are formed by interactions between proteins including tetraspanins (Yanez-Mo et al., 2009). TEMs act as interacting platforms that aid the selection of surface and intracellular proteins to be sorted toward exosomes (Perez-Hernandez et al., 2013) . A typical tetraspanin contains four transmembrane domains connected by small intracellular loops as well by a small extracellular loop (SEL) and a large extracellular loop (LEL) (Boucheix and Rubinstein, 2001; Maecker et al., 1997) . Tetraspanins are further defined by conserved amino acid sequences among the highly conserved CCG motif. The LEL is of special interest because it is important for protein-protein interactions (PPI), e.g., with viral proteins (Ivanusic et al., 2021a; Rajesh et al., 2012) . To investigate the influence of the LEL for recruiting CD63 to exosomes, different constructs were employed ( **Figure 1A** ). First, the full-length CD63 sequence containing amino acids (aa) 1–238 fused to the mCherry protein (pCMV-CD63-mCherry). Second, CD63 with deleted LEL (deleted aa 111–200) and containing the V5-6xHis-tag that connects the CD63 transmembrane domain 3 and 4 (pCMV-CD63ΔLEL-V5-His-mCherry). Third, the V5-6xHis-tag was introduced between aa 133 and 134 (pCMV-CD63-LEL-V5-His-mCherry). The region where the linker V5-6xHis was introduced contains variable sites that are important for protein-protein interaction with other membrane proteins. This site will not influence conserved regions that are important for homodimerization within the tetraspanin enriched microdomains (Hemler, 2005; Seigneuret et al., 2001; Stickney et al., 2016; Stipp et al., 2003; Yánez-Mó et al., 2001). In contrast to the construct with the deleted LEL this construct contained all amino acids of the CD63 LEL. As mentioned, all constructs contained N-terminally a FLAG-tag and were fused C-terminally with the mCherry sequence as a fluorescent marker protein to localize and quantify expressed proteins. The construct pmCherry-N1 expressed the monomeric mCherry protein without a FLAG-tag and the V5-tag was used as a control. To follow up the fate of expressed proteins with different CD63 LEL content, HEK293T cells were transfected with the generated constructs and exosomes were isolated from the collected supernatant. Exosomes and cell lysates were analyzed in parallel. The FLAG-tag was found in the exosomes only when the CD63LEL was present in the construct transfected to the HEK293T cells ( **Figure 1B** ). When the PVDF membrane was reprobed using antibodies against the V5-tag, a very strong band was only observed when the construct was transfected in which the V5-His-tag was introduced into the CD63 LEL, but not when the V5-tag was replaced against the LEL. In this case only a faint band was observed ( **Figure 1B** ). This suggests that the CD63 LEL was important for directing the CD63 fused with mCherry into exosomes. Probing with antibodies against CD9 and CD81 showed that both proteins were present in the exosome preparation. This demonstrated that the preparation contained exosomes as described (Fordjour et al., 2022) . Quantification of mCherry showed that the expression levels of the proteins CD63-mCherry, CD63ΔLEL-V5-His-mCherry, and CD63-LEL-V5-His-mCherry during the experiments were at the same level. Therefore, a reduced concentration or even absence of the protein CD63ΔLEL-V5-His-mCherry can be excluded ( **Figure 1C** ). Next, the distribution of CD63 proteins fused with mCherry were compared to the CD63-YFP protein which was co-transfected into the HEK293T cells ( **Figure 1D** ). For this, the Pearson correlation coefficient (PCC) was calculated. The PCC for the expressed protein CD63ΔLEL-V5-His-mCherry was significantly lower than for CD63-LEL-V5-His-mCherry in correlation to CD63-YFP ( **Figure 1E** ). However, the correlation of CD63ΔLEL-V5-His-mCherry with CD63-YFP achieved a mean value of *r* = 0.69, which is still considered a strong correlation. In contrast, the protein CD63-LEL-V5-His-mCherry showed a very strong correlation ( *r* = 0.90) with CD63-YFP within the cell. The localization of CD63ΔLEL-V5-His-mCherry and CD63-LEL-V5-His-mCherry in the plasma membrane was demonstrated by analyzing the accessibility of anti-V5 antibodies against the exposed V5-epitope. Anti-V5 antibodies were able to bind to the V5-epitop of both proteins, indicating a localization in the plasma membrane. Furthermore, no differences in the distribution of CD63ΔLEL-V5-His-mCherry and CD63-LEL-V5-His-mCherry in the plasma membrane were observed. This means that the protein CD63ΔLEL-V5-His-mCherry is accumulating on the cell surface but is not recruited to the exosomes. This observation is important because CD63 at the cell surface may be endocytosed into clathrin-coated vesicles then delivered to early endosomes (Hunziker and Geuze, 1996; Kobayashi et al., 2000) . This led to the enrichment of CD63 in late endosomal and lysosomal compartments following accumulation in intraluminal vesicles that bud into MVBs (Pols and Klumperman, 2009) . MVBs then fuse with the plasma membrane and exosomes are released from the cell (Xu et al., 2022) . Taken together, we suggest that CD63 without LEL content is not able to be secreted into exosomes although the protein is expressed on the cell surface. The higher intracellular distribution of CD63ΔLEL-V5-His-mCherry suggests that there is a false sorting between the plasma membrane and MVB. If CD63ΔLEL-V5-His-mCherry reaches MVBs it should be detectable in isolated exosomes. Further experiments are needed to investigate how the LEL contributes to the insertion of CD63 into exosomes. We assume that the loss of lateral interaction with the TEMs could be a reason that CD63 without LEL is not released by exosomes. Furthermore, it would be very interesting to see that the LEL may be sufficient to recruit heterologous proteins specifically into exosomes.

## Methods

**Molecular cloning**

The construction of the expression constructs pCMV-CD63-mCherry, pCMV-CD63-YFP, and pCMV-CD63ΔLEL-V5-His-mCherry were previously described (Ivanusic et al., 2016; Ivanusic et al., 2021b). The construct pCMV-CD63-LEL-V5-His-mCherry was generated by introducing a gen synthesis fragment coding for the CD63 amino acids (aa) 1–133 and 134–238 (GenBank accession AHI51903.1) connected by a V5-6xHis linker, V5 was inserted in the same way as described for pCMV-CD63ΔLEL-V5-His-mCherry . The fragment was cloned using the restriction sites *Not* I/ *Xho* I (New England Biolabs, NEB, Frankfurt, Germany) in the pCMV-CD63-mCherry vector. The fragment was ligated using the T4 ligase (NEB) and transformed into *E. coli* DH5α competent cells (NEB). The resulting construct was confirmed by restriction analysis and Sanger sequencing.

**Cell culture and transfection**

HEK293T cells were maintained in Dulbecco's modified Eagle's medium (DMEM) supplemented with 10% fetal bovine serum (Gibco, Thermo Fisher, Schwerte, Germany), 100 IU/mL penicillin, 100 µg/mL streptomycin (Gibco), and 2 mM L-glutamine (Gibco) at 37 °C, 5% CO _2_ , and a relative humidity of about 95%. 3.0 x10 ^5^ (6-well format) HEK293T cells were seeded and transfected at a confluency of 80–90% with 2 µg of indicated plasmid DNA diluted in 200 µl Jetprime buffer and 4 µl Jetprime reagent (Polyplus, Illkirch, France) according to the manufacturer’s instructions. Transfection mix was added to the cells and incubated for 48 h.

**Isolation of exosomes**

2 ml of collected cell culture supernatant from transfected HEK293T cells were processed with the Exosome Isolation Kit CD9, human (Miltenyi Biotec, Bergisch Gladbach, Germany) according to the manufacturer’s instructions, including filtration through a 0.2 µm Minisart syringe filter (Sartorius, Göttingen, Germany). In the last step the exosomes were elutated with 50 µl of 2x Laemmli buffer (Sigma Aldrich, Darmstadt, Germany) from the magnetic column.

**Western Blot Analysis**

HEK293T cells were lysed after 48 h post-transfection in 200 µl 2x ready to use Laemmli buffer (Sigma) containing 200 U/ml Benzonase (Merck, Darmstadt, Germany). Samples were heated for 3 min at 90 °C and 10 µl were separated using a Mini-PROTEAN TGX precast gel (BioRad, Munich, Germany) and 1x running buffer (10x buffer contains 144.4 g glycine, 30.3 g Tris base, 10 g SDS in 1 L). Separated proteins were blotted onto a 0.2 µm Trans-Blot PVDF membrane (BioRad) and blocked in 10% low fat milk powder (Carl Roth, Karlsruhe, Germany) for 45 min. Lysates from the exosome fraction were processed for Western blot analysis in the same way. The membrane was probed after blocking with indicated antibodies anti-FLAG-HRP dilution 1:10,000 (Thermo Fisher Scientific), mouse anti-V5-HRP dilution 1:10,000 (Thermo Fisher Scientific), mouse anti-CD9 dilution 1:3,000 (Thermo Fisher Scientific), rabbit anti-CD81 dilution 1:2,000, and mouse anti-actin-beta (ACTB)-HRP dilution 1:8,000 (Thermo Fisher Scientific) for 1 h. Primary antibodies were detected by incubation for 45 min with anti-mouse-HRP (DAKO, Hamburg, Germany) or anti-rabbit-HRP (DAKO) at a dilution of 1:10,000. HRP-activity was detected by incubation with SuperSignal West Pico PLUS or SuperSignal West Atto chemiluminescent substrate (Thermo Fisher Scientific) and monitored with a ChemoCam device (Intas, Göttingen, Germany). For re-probing of PVDF membrane they were incubated two times for 10 min with stripping buffer containing 15 g glycine (Carl Roth), 1 g SDS (Carl Roth), and 10 ml Tween (Sigma) per 1 L, pH-value of 2.2.

**Quantification of mCherry protein**

For quantification of mCherry protein the mCherry quantification kit was utilized (Abcam, Netherlands). In detail, HEK293T cells were lysed in 1 ml mCherry assay buffer, frozen, thawed, and centrifuged to pellet the cell debris. Supernatants were collected and 100 ul were placed in an optical plate (BioGreiner One, Frickenhausen, Germany). A mCherry standard curve was prepared as described in the manual and the fluorescence intensity was assayed with the Promega GloMax device with filters for red dyes. The supernatant containing mCherry was diluted 1:10 to achieve the same fluorescence intensity range as the other samples. The amount of mCherry protein from supernatants was calculated using data from the standard curve.

**Analysis of extracellular accessibility of the V5-epitope and colocalization by confocal laser microscopy (cLSM)**

HEK293T cells (1 × 10 ^4^ /well) were seeded into a high glass-bottomed eight well IBIDI μ-slide (Ibidi, Munich, Germany), and after 24 h cells were transiently transfected with 0.1 μg plasmid DNA diluted in 10 μl Jetprime buffer and 0.2 μl Jetprime transfection reagent. In case of co-transfection, we used 0.4 μl Jetprime transfection reagent. After 48 h, cells were washed once with 1 × phosphate-buffered saline (PBS) and fixed with 200 μl of 2% paraformaldehyde in 1 × PBS for 20 min. Cells were washed two times with 200 μl 1 × PBS and were leaved in 200 μl blocking buffer containing 10% normal goat serum. Rabbit anti-V5 antibody (Novus Biologicals, Littleton, USA) was diluted (1:2,000) in blocking buffer containing 10% normal goat serum and 200 μl was added to the cells, incubated for 1.5 h at room temperature, washed three times with 1 × PBS, and incubated with anti-rabbit-Alexa 488 antibody (Thermo Fisher Scientific) in a dilution of 1:3,000 in 3% BSA for 1 h. After three washing steps cells were left in 200 μl 1 × PBS in the presence of 1 μl of Hoechst 33342 staining solution (Immunochemistry technologies, CA, USA). Images were acquired using an inverted confocal laser scanning microscope ZEISS Axioscope 780 (Zeiss, Oberkochen, Germany) and a plan-apochromat oil immersion objective 63 ×, N.A. 1.4. Fluorescence signals were detected with the Zeiss ZEN smart setup settings for mCherry, Hoechst 33342, and Alexa 488 dyes. In the case colocalization analysis, HEK293T cells were transfected with indicated plasmid DNA and imaged using the setting for mCherry, Hoechst 33342, and YFP. PCC was calculated using the coloc2 plugin from the Fiji image software (Schindelin et al., 2012) .

## Reagents

**Gen synthesis fragment for construction of pCMV-CD63-LEL-V5-His-mCherry**

gagctccaccgcggtggcggccgccaccatggattacaaggatgacgacgataaaggcccgggcggatccatggcggtggaaggaggaatgaaatgtgtgaagttcttgctctacgtcctcctgctggccttttgcgcctgtgcagtgggactgattgccgtgggtgtcggggcacagcttgtcctgagtcagaccataatccagggggctacccctggctctctgttgccagtggtcatcatcgcagtgggtgtcttcctcttcctggtggcttttgtgggctgctgcggggcctgcaaggagaactattgtcttatgatcacgtttgccatctttctgtctcttatcatgttggtggaggtggccgcagccattgctggctatgtgtttagagataaggtgatgtcagagtttaataacaacttccggcagcagatggagaattacccgaaaaacaaccacactgctggaaagggcccgcggttcgaaggtaagcctatccctaaccctctcctcggtctcgattctacgcgtaccggtcatcatcaccatcaccattcgatcctggacaggatgcaggcagattttaagtgctgtggggctgctaactacacagattgggagaaaatcccttccatgtcgaagaaccgagtccccgactcctgctgcattgatgttactgtgggctgtgggattaatttcaacgagaaggcgatccataaggagggctgtgtggagaagattgggggctggctgaggaaaaatgtgctggtggtagctgcagcagcccttggaattgcttttgtcgaggttttgggaattgtctttgcctgctgcctcgtgaagagtatcagaagtggctacgaggtgatgaagcttatcgataccgtcgacctcgag
